# Structure guided design of an antibacterial peptide that targets UDP-*N*-acetylglucosamine acyltransferase

**DOI:** 10.1038/s41598-019-40418-8

**Published:** 2019-03-08

**Authors:** Manchuta Dangkulwanich, Christian R. H. Raetz, Allison H. Williams

**Affiliations:** 1 Department of Biochemistry, Duke University Medical Center, Box 3711, Durham, North Carolina 27710 USA; 2 Science Division, Mahidol University International College, Mahidol University, Phutthamonthon 4 Road, Salaya, Nakhon Pathom 73170 Thailand; 3 Institut Pasteur, Département de Microbiologie, Unité Biologie et Génétique de la Paroi Bactérienne, 28 Rue du Dr. Roux, 75015 Paris, France

## Abstract

UDP-*N*-acetylglucosamine (UDP-GlcNAc) acyltransferase (LpxA) catalyzes the first step of lipid A biosynthesis, the transfer of an *R*-3-hydroxyacyl chain from its acyl carrier protein (ACP) to the 3-OH group of UDP-GlcNAc. Essential in the growth of Gram-negative bacteria, LpxA is a logical target for antibiotics design. A pentadecapeptide (Peptide 920) with high affinity towards LpxA was previously identified in a phage display library. Here we created a small library of systematically designed peptides with the length of four to thirteen amino acids using Peptide 920 as a scaffold. The concentrations of these peptides at which 50% of LpxA is inhibited (IC_50_) range from 50 nM to >100 μM. We determined the crystal structure of *E. coli* LpxA in a complex with a potent inhibitor. LpxA-inhibitor interaction, solvent model and all contributing factors to inhibitor efficacy were well resolved. The peptide primarily occludes the ACP binding site of LpxA. Interactions between LpxA and the inhibitor are different from those in the structure of Peptide 920. The inhibitory peptide library and the crystal structure of inhibitor-bound LpxA described here may further assist in the rational design of inhibitors with antimicrobial activity that target LpxA and potentially other acyltransferases.

## Introduction

Lipid A is the hydrophobic anchor that secures the sugar components (core and O-groups) of lipopolysaccharide (LPS) to the external surface of the outer membrane^1,2^. The lipid A component of LPS also elicits an immune response in animal systems^2–4^. The minimal structure required for the viability of Gram-negative bacteria is lipid IV_A_^5^. Because lipid A is essential for the viability of Gram-negative bacteria, all the enzymes involved in its biosynthesis represent potential targets for inhibitory compounds with antibacterial activities^6^.

UDP-*N*-acetylglucosamine acyltransferase (LpxA) catalyzes the first step of lipid A biosynthesis^2,3^. LpxA catalyzes the transfer of an *R*-3-hydroxymyristoyl chain from ACP to the 3-OH glucosamine group of UDP-GlcNAc. The acylation of UDP-GlcNAc is a thermodynamically unfavorable reaction with an equilibrium constant of ~0.01^7,8^. Consequently, the second step of lipid A biosynthesis, catalyzed by the deacetylase LpxC, is the committed and first irreversible step of this pathway. Lipid A synthesized by most Gram-negative bacteria is similar to that of *E. coli*. Structural differences observed in the lengths of the acyl chain in the lipid A of various Gram-negative bacteria at the 3 and 3′ positions of the disaccharide glucosamine ring can be attributed to the differences in acyl chain specificity of the various LpxA orthologs. LpxA shows specificity for acyl chain containing a 3-OH moiety and only utilizes ACP as its donor substrate^7^. For example, *E. coli* LpxA is unable to substitute myristoyl-ACP for *R*-3-hydroxymyristoyl-ACP^7^. However, myristoyl-ACP appears to be an inhibitor of LpxA. Interestingly, *Chlamydia trachomatis* LpxA is unique in its ability to utilize myristoyl-ACP instead of *R*-3-hydroxymyristoyl-ACP^9^. Though highly specific for a *R-*3-hydroxymyristoyl-ACP, *E. coli* LpxA is capable of utilizing a shorter *R*-3-hydroxydecanoyl-ACP and *R*-3-hydroxylauroyl-ACP, albeit far less efficiently^7,10^. A single amino acid change in *E. coli* LxpA appears to be responsible for acyl chain specificity. A G173M mutation reverses the acyl chain specificity of *E. coli* LpxA from a 14-carbon acyl chain dependent enzyme to a 10-carbon acyl chain dependent enzyme^11^. The reverse mutation (M169G) in *Pseudomonas aeruginosa*, LpxA converts the acyl chain preference from a 10-carbon acyl chain to a 14-carbon acyl chain^11,12^.

The crystal structure of LpxA reveals a homotrimer that possesses a left-handed parallel β-helix. Each monomeric subunit of the trimer contains two domains, an NH_2_-terminal β-helical domain (residues 1–186), and a COOH-terminal α-helical domain (residues 187–262)^13–15^. The active site is located in a deep cleft between adjacent subunits where there are multiple conserved histidines and other basic residues. Each active site is related by symmetry, and therefore LpxA has three identical active sites. Previous crystallographic and mutagenesis studies demonstrated that LpxA’s likely proceed through a general base catalytic mechanism using an absolutely conserved catalytic histidine (H125)^13,14,16^. Other conserved basic residues such as H122, H144, H160, and R208 were proposed to be involved in substrate binding based on mutagenesis studies that revealed a significant reduction in the enzymes’ activities as well as a shift in the apparent *K*_m_s^13,14,16^.

Previously, we described Peptide 920, a 15 amino acid long potent inhibitor of *E. coli* LpxA with an IC_50_ of 60 nM^13^. Using Peptide 920 as a scaffold, a small peptide library was created and screened. Our goal was to search for a smaller efficacious peptide with an enhanced inhibitory potential, as well as to identify the essential residues that contribute to inhibitor potency (Table 1). The most potent inhibitor was structurally characterized to visualize carefully those interactions that contributes to inhibitor efficacy.

**Table 1:** A small library of the truncated Peptide 920 and their IC_50_.

Peptide identity	Peptide sequence	IC_50_
Peptide 920	SSGWMLDPIAGKWSR	60 ± 9 nM*
*Truncation of Peptide 920 N-terminus*		
*CR19*	GWMLDPIAGKWSR	77 ± 3 nM
*CR20*	WMLDPIAGKWSR	50 ± 6 nM
*Truncation of Peptide 920 C-terminus*		
*CR21*	SSGWMLDPIAGKWS	12 ± 1.4 μM
*CR22*	SSGWMLDPIAGKW	9 ± 2.2 μM
*Truncation of Peptide 920 N and C- terminus*		
*CR23*	GWMLDPIAGKW	10 ± 0.3 μM
*CR24*	GWMLDPIA	>100 μM
*CR25*	WMLDPI	>100 μM
*CR26*	WMLD	>100 μM

^*^IC_50_ value taken from previous publication^13^. Error bars show standard deviations of triplicates.

Here we report the successful crystallization of *E. coli* LpxA complexed with peptide CR20 (WMLDPIAGKWSR) at a resolution of 1.60 Å. Peptide CR20 is a potent inhibitor of *E. coli* LpxA with an IC_50_ of ~50 nM. The peptide is located at the interface of each adjacent subunit and interacts with residues from both sides. It occupies part of the ACP binding site that was inferred from previous structural and mutagenesis studies^13,17^. All the residues of the peptide CR20 were well resolved. The design and characterization of our small library of peptides complemented with the structural characterization of LpxA-peptide CR20 complex sheds light on the key residues in LpxA that are important inhibitory targets. It also shed light on the residues of the peptide that contributes to inhibitor efficacy. For example, removing a single amino acid from the C-terminus of the peptide results in a three orders of magnitude loss in the efficacy of peptide CR20. Additionally, a higher resolution the crystal structure of peptide CR20 bound to LpxA may assist in the rational design of inhibitors with antibiotic activity.

## Results

### A small library of truncated peptides

Peptide 920 was identified in phage display library, and when expressed fused to glutathione sepharose (GST) in *E. coli*, it inhibited bacterial growth and showed high specificity for its target, LpxA^18^. Here we systematically designed and synthesized a small library of peptides using Peptide 920 as the scaffold. The peptides were assayed for acyl transferase inhibitory activity under conditions where the concentrations of UDP-GlcNAc and *R*-3-hydroxymyristoyl-ACP were 1 µM each. The data revealed that truncations at the *N*-terminus of Peptide 920 were tolerated more so than truncations at the *C*-terminus (Table 1). Removal of three residues at the N-terminal of Peptide 920 created an efficacious peptide (CR20) with an IC_50_ ~ 50 nM. Conversely, removal of the arginine from the *C*-terminus (Peptide CR21) resulted in a two orders magnitude shift in the IC_50_ (10 μM). Peptide CR21 and CR23 still have good inhibitory potential of 10 μM albeit lower than peptide CR20. This data suggests the contacts made by the *C-*terminus of the peptide are crucial for inhibitor efficacy and probably stability in the active site of LpxA.

**Figure 1:**
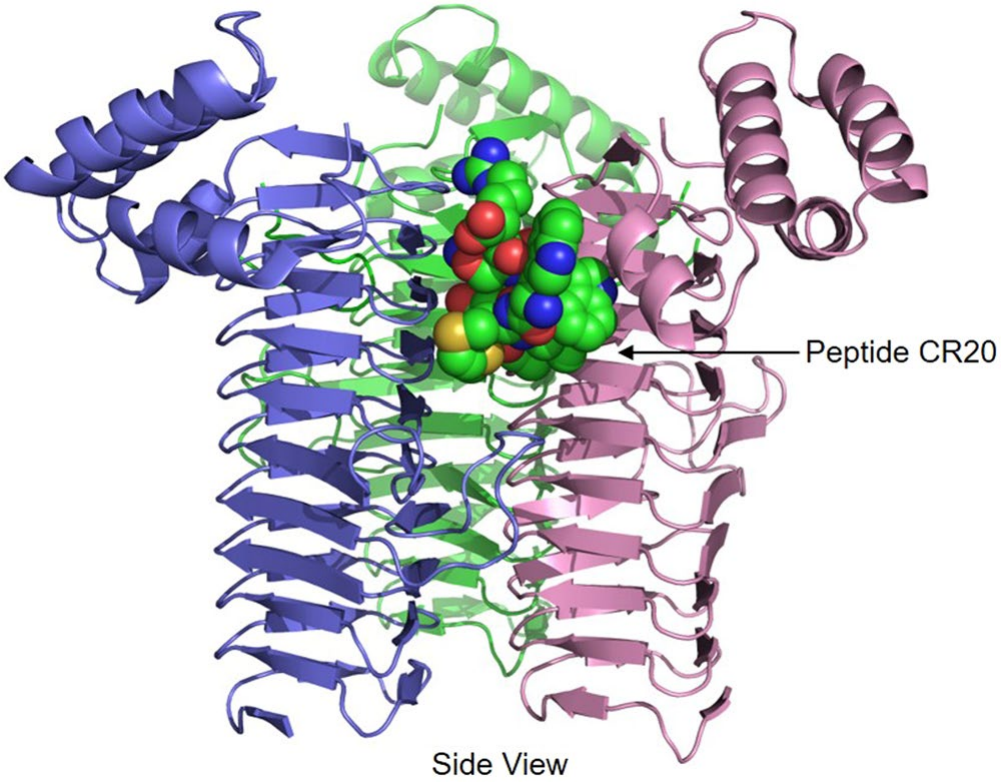
Crystal Structure of LpxA-peptide CR20 complex at 1.60 Å. The individual subunits are colored pink, green, and blue. The LpxA *N* termini is located at the bottom, and displays the start of the β-helix domain of each subunit. The peptide (green) is in a β-hairpin conformation with the *N* and *C* termini exposed to solvent. In the peptide, the carbons are colored green, the nitrogens blue, and the oxygens red. The free enzyme (LpxA)and LpxA-peptide CR20 reveal that there were not much global movements in the side chains except for minor perturbations of those interacting directly with the peptide. LpxA appears to be a rigid structure where bound ligands are more likely to adopt different conformations, as noted previously^13^.

**Figure 2:**
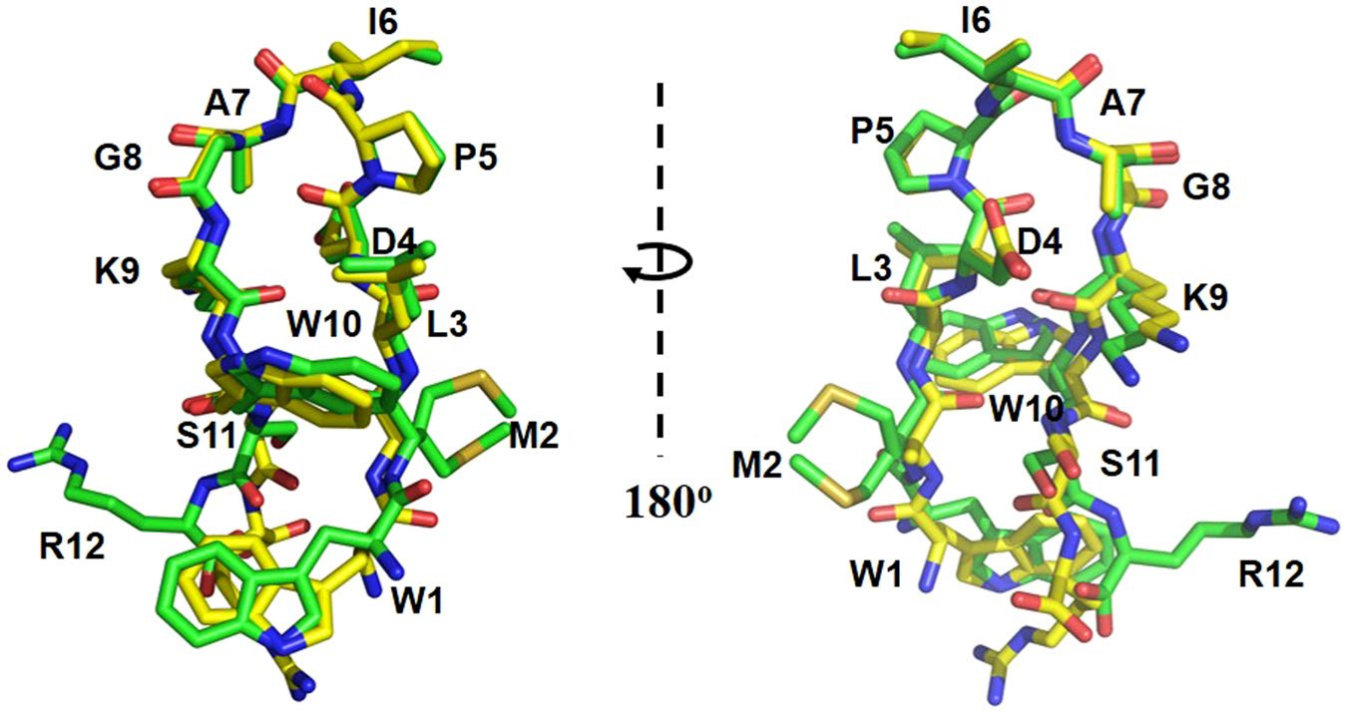
Alignment of Peptide 920 and peptide CR20. The two peptides fold slightly different. Peptide 920 (PDB ID: 2AQ9) carbons are colored in yellow, oxygens in red and nitrogens in blue. Peptide CR20 carbons are colored in green, oxygens in red and nitrogens in blue. The model on the right is rotated 180°, showing the rear view of the model on the left.

To structurally assess the specific interactions of peptide CR20 with LpxA, this complex was crystallized. LpxA was crystallized in the presence of 25 molar excess of peptide CR20. The crystals diffracted to a resolution of 1.60 Å and the complex solved by molecular replacement using the coordinates of the free enzyme (PDB ID: 1LXA) as a model. There is a single LpxA molecule in the asymmetric unit, and three asymmetric units associate around a crystallographic three-fold axis to form the homotrimer of LpxA (Fig. 1). The refinement converged R_work_ and R_free_ to 0.191 and 0.218 respectively, (Table 2) with a low all-atom clash score and good geometry. All 262 residues of *E. coli* LpxA were visible, with the exception of the methionine side chain at position one. All 12 residues of the peptide were clear and well defined with multiple conformations of the side chain of the methionine at position 2 (Fig. 2).

**Table 2:** Data-collection and refinement statistics. Values in parentheses are for the outer shell.

Data Collection	LpxA
Ligand added	Peptide CR20
**Data collection**	
Wavelength (Å)	1.5415
Resolution range (Å)	21.65–1.60 (1.69–1.60)
Space group	P2_1_3
*a* = *b* = *c* (Å)	96.73
CC1/2 (%)	94.80 (76.70)
Unique reflections	34668 (2647)
Completeness (%)	99.84 (100)
Mean *I*/σ(*I*)	14.2(3.9)
Wilson B factor (Å^2^)	23.684
*R*_merge_^a^	0.078 (0.085)
**Refinement**	
*R*_factor_^b^	0.1910 (0.453)
*R*_free_^c^	0.2260 (0.3850)
No. of atoms	2500
No. of waters	400
No. of protein residues	274
R.m.s.d., bonds (Å)	0.007
R.m.s.d., angles (°)	1.091
Ramachandran favored (%)	98.5
Ramachandran outliers (%)	0
***B*** **factors (Å** ^**2**^ **)**	
Protein	23.68
Ligand	25.68
Solvent	38.90
All-atom clash score	2.85
Molprobity score	1.07 (99th percentile)

Resolution limit was defined as the highest resolution shell where the average I/σ_(I)_ was 2.

^a^R_merge_ = Σ_hkl_Σ_i_|I_i(hkl)_ − <I_(hkl)_>|/Σ_hkl_Σ_i_I_(hkl)_.

^b^R_factor_ = Σ|F_o_ − F_c_|/ΣF_o_. Five percent of the reflections was used to calculate ^c^R_free_.

Peptide CR20 residues bind between adjacent subunits in the active site region. The left-handed β-helix fold of LpxA is not perturbed upon peptide binding (Fig. 1). An overlay of LpxA-Peptide 920^13^ and LpxA-peptide CR20 gave a root-mean-square distance of 0.074 for all Cα pairs of the backbone. A comparison of the side chains from the free enzyme (LpxA), and LpxA-peptide CR20 reveal that there were not much global movements in the side chains or backbone of LpxA except for minor perturbations of those interacting directly with the peptide^15^. LpxA is a rigid structure where bound ligands are more likely to adopt different conformations.

### Interactions between peptide CR20 and LpxA

LpxA-bound peptide CR20 is folded into a β-hairpin conformation. The hairpin inserts into the active site of LpxA and the *N* and *C* termini of the peptide is solvent exposed. Peptide CR20 makes extensive contacts with LpxA. An overlay of Peptide 920 and peptide CR20 reveals similarity between the buried portions of the peptide; however, there were differences observed in the residues at the end of the *C* and *N* termini (Fig. 2)^13^. In LpxA- Peptide 920 there appears to be a cation-π interaction between the arginine (R15) at the *C*-terminus of the peptide and the tryptophan (W4) at the *N*-terminus. In contrast, the R15 in peptide CR20, is flipped 180°, (Fig. 2) while, the tryptophan is interacting with R204 via a water-mediated interaction instead of a cation-π interaction (Fig. 2). This observation suggests that these bulky residues are indeed flexible and they could be manipulated for peptidomimetics.

**Figure 3:**
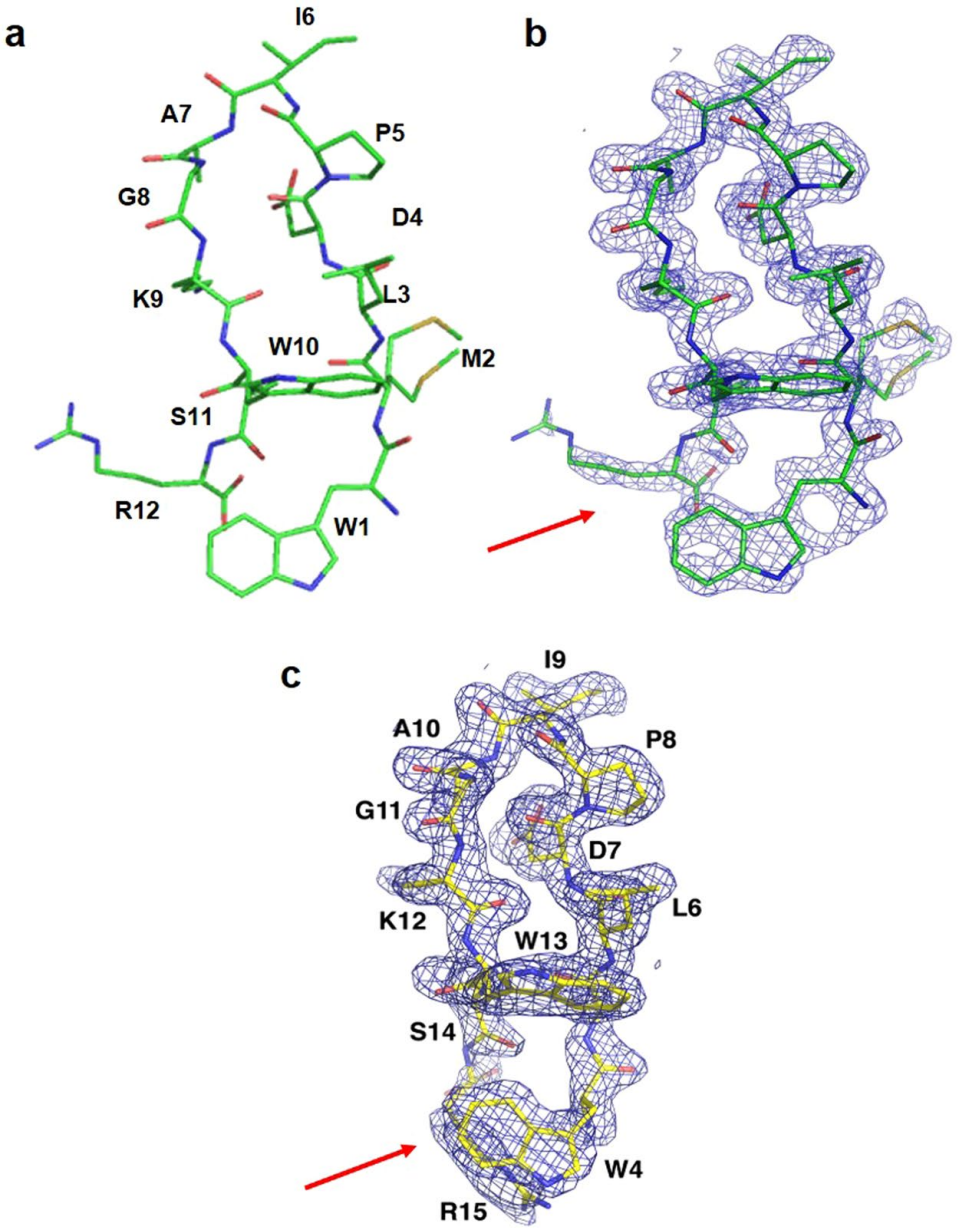
Electron density maps of peptide CR20 and Peptide 920. **(a)** Peptide CR20 is shown as a stick model with carbons in green. **(b)** The 2fo-fc electron density map is contoured at 1σ around peptide CR20. There are multiple conformations of the methionine side chain. **(c)** The 2fo-fc electron density map contoured at 1σ around Peptide 920 (PDB ID: 2AQ9). The density for the methionine side chain was missing in this model. Red arrows point to the different orientations in residues at the *N* and *C* termini of the two peptides.

**Figure 4:**
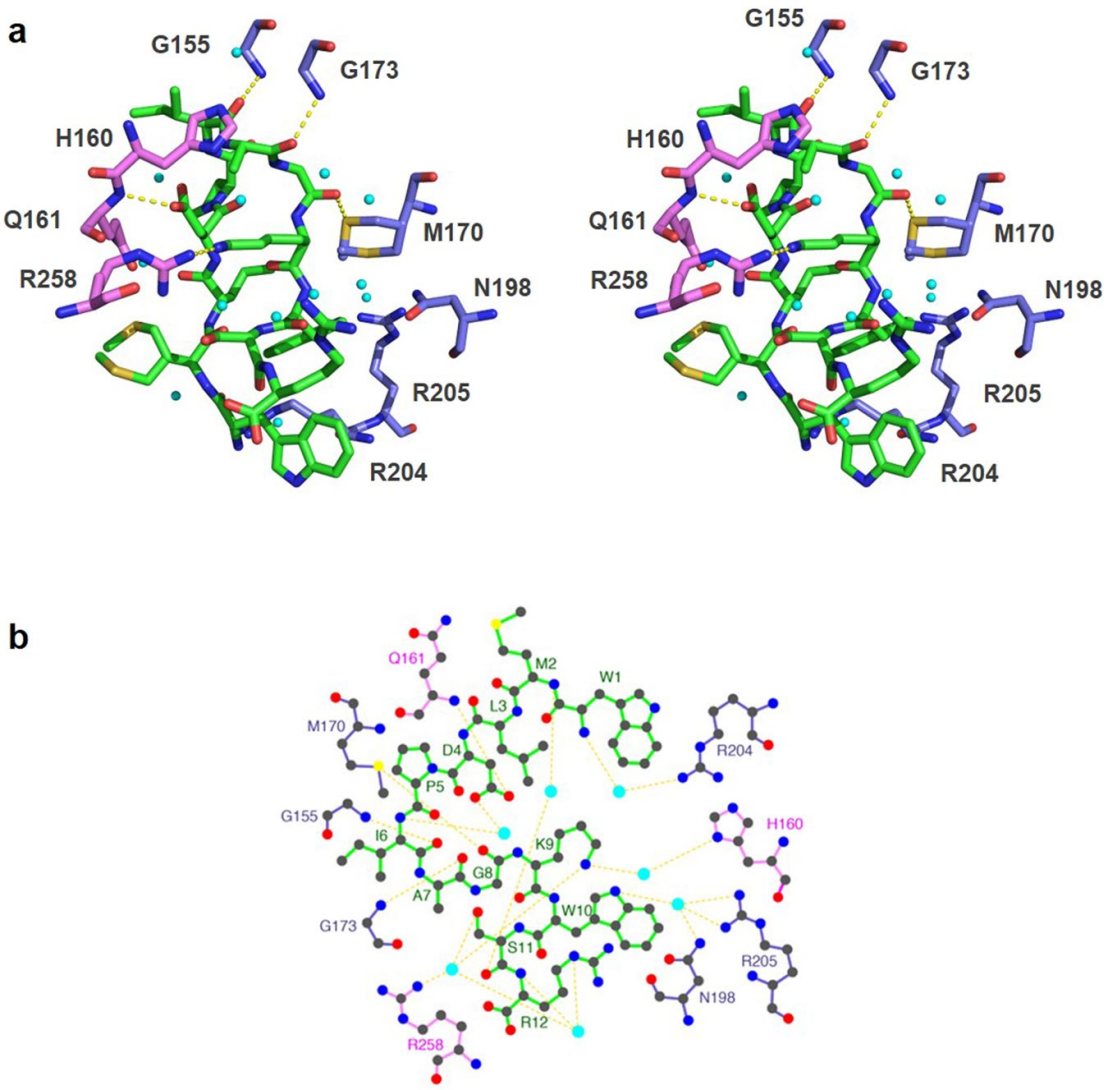
A close-up of peptide CR20 interactions with LpxA. **(a)** Stereo-view of polar interactions made by LpxA-peptide CR20. Peptide CR20 is colored in green and the LpxA subunit is colored in blue or pink, consistent with the color scheme of Fig. 1. All the N and O atoms are blue and red, respectively. Hydrogen bonds are yellow dashed lines. Water molecules are cyan spheres. Hydrogen bonds involving water are not included for clarity. **(b)** Key interactions between peptide CR20 and LpxA in 2D. Hydrogen bonds are shown as yellow dashes. Water molecules are cyan circles. The 2D interaction diagram was created by LigPlot + software^29^.

The overall density of the peptide CR20 (all 12 residues) and the surrounding residues from LpxA was clear and continuous (Fig. 3). Peptide CR20 does not disrupt any contacts between the individual monomers of LpxA, but instead forms a bridge between the adjacent subunits. The significant interaction of peptide CR20 with LpxA is sustained by hydrogen bonds, some of which are mediated by water molecules. There is indeed an intricate hydrogen-bonding network with at least thirteen water molecules that are directly hydrogen-bonded to peptide CR20 (Fig. 4). Residues of LpxA involved in direct hydrogen bonds with the peptide are Q161 (backbone N) and R258 from one protomer and G155, G173, and M170 from the adjacent protomer (Fig. 4). These direct hydrogen bonds are also present in the LpxA-Peptide 920 complex^13^. However, there were differences, in the partners they engage with for hydrogen bond interactions. For example, R258 is directly hydrogen bonded to K9 of peptide CR20 instead of S11, and M170 is hydrogen bonded to the backbone carbonyl of G8 in peptide CR20 instead of W10 (Fig. 4). Residues that interact with peptide CR20 via water-mediated interactions are R204, R205, N198, and Q161 (side chain) from the blue chain and H160 from the pink chain. Only, Q161 water mediated interaction was previously observed in the LpxA-Peptide 920 complex^13^ (Fig. 4). It appears that there are localized deviations in the way the peptide is supported in the active site of LpxA, although peptide-binding region remains the same. There are four additional hydrogen bonds that are formed within peptide CR20. The peptide β-hairpin conformation may be supported by these intra-peptide hydrogen bonds.

### The importance of residues implicated in peptide binding

**Figure 5:**
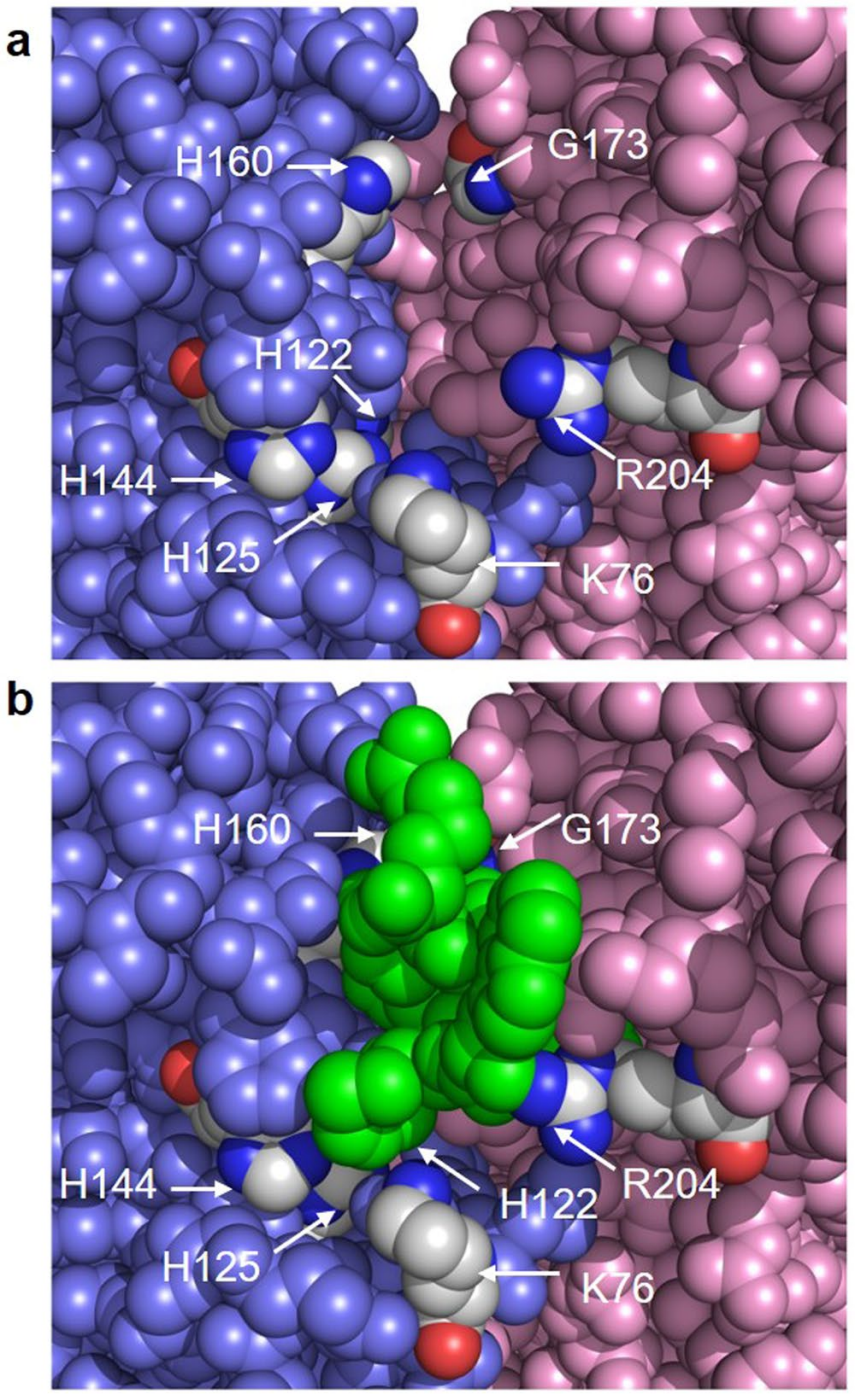
Location of peptide CR20 and conserved residues implicated in catalysis. **(a)** View into one of the three peptide-binding cavities of the LpxA trimer with the peptide CR20 hidden. LpxA subunits are colored pink, slate, or green (not visible) as in Fig. 1. Key residues implicated in catalysis or substrate binding by mutagenesis are colored according to element with carbons in grey. **(b)** Same view as above but with peptide CR20 present in green. Conserved LpxA residues are labeled in white. There is some space between peptide CR20 and the conserved residues H125, H144, H122 and K76. Peptide CR20 is interacting with G173 and H160, these residues are largely hidden from view. In the complete model, a few water molecules are located in the space beneath peptide CR20 (not shown).

Peptide CR20 is interacting with residues implicated in substrate binding as revealed by previous mutagenesis studies (H160, G173 and R204) (Fig. 5)^19^. G173 involved in acyl chain binding is not interacting with the proposed catalytic residue (H125) but could potentially block or partially occlude access to this residue. The peptide interacts with H160 via a water-mediated interaction (Fig. 4). The H160A mutant has significantly less activity than the wild-type^19^ however this residual activity was resistant to inhibition by peptide CR20 (IC_50_ ~10,000 µM) (data not shown). This was surprising at first, however, H160 is interacting with K9 of the C-terminus of the peptide CR20, which is essential to the efficacy of the peptide interaction with LpxA.

## Discussion

Lipid A (endotoxin) plays an important role in bacterial growth, outer membrane integrity, and stimulation of the mammalian immune system^1,2,20^. All the enzymes in the lipid A pathway are potential targets for inhibitors and some may be effective antibiotics. LpxA catalyzes the first step of lipid A biosynthesis and is essential for viability in most Gram-negative pathogens^2^.

**Figure 6:**
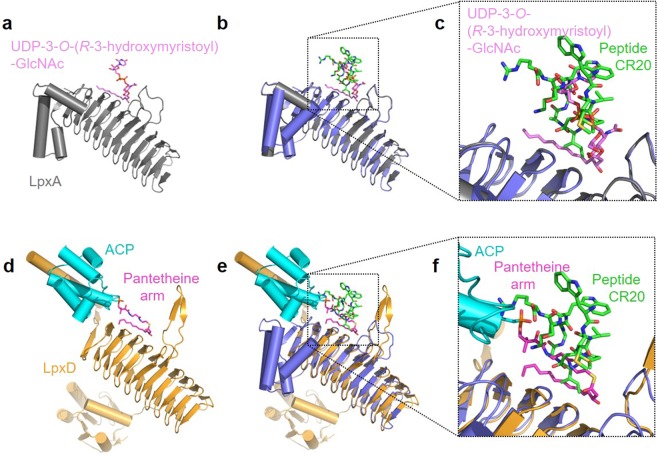
Superpositions of LpxA and LpxD. **(a)** The structure of LpxA with the bound product, UDP-3-*O-*(*R*-hydroxymyristoyl)-GlcNAc (PDB ID: 2QIA). LpxA is shown in grey and UDP-3-*O-*(*R*-hydroxymyristoyl)-GlcNAc is shown in pink. **(b)** LpxA-peptide CR20 superposed with the product bound structure of LpxA. **(c)** A close-up view to the product binding site. Peptide CR20 overlaps with UDP-3-*O-*(*R*-hydroxymyristoyl)-GlcNAc product. **(d)** The structure of LpxD in complex with the intact *R*-3-hydroxymyristoy-ACP (PDB ID: 4IHF). LpxD is shown in orange. ACP and the pantetheine arm are shown in cyan and magenta, respectively. **(e)** LpxA-peptide CR20 superposed with LpxD and intact *R*-3-hydroxymyristoy-ACP. **(f)** A close-up view to the acyl-ACP binding site. Peptide CR20 occludes the binding site of acyl-ACP at the pantetheine arm.

The *E. coli* LxpA-peptide CR20 complex solved at high resolution (1.60 Å) facilitated the identification of a dedicated space in the essential LpxA enzyme that can be targeted for inhibitors. LpxA has two substrates: UDP-GlcNAc and acyl-ACP. The binding site of both substrates have been identified in previous structural studies^13,14,16,17^. Peptides CR19-CR22 represent potential starting points for the design of potent more efficacious inhibitor that target LpxA (Table 1). The most efficacious peptide CR20 binds in a region that would mainly occlude the binding site of acyl-ACP. The superposition of the CR20-bound LpxA to the UDP-3-*O-*(*R*-hydroxymyristoyl)-GlcNAc product bound structure shows that peptide CR20 overlaps with the UDP-3-*O-*(*R*-hydroxymyristoyl)-GlcNAc product in active site of LpxA (Fig. 6a–c). Peptide CR20 is a potent inhibitor of LpxA with an IC_50_ of approximately 50 nM (Table 1).

Peptide CR20 is more ordered in the active site of LpxA than Peptide 920. The electron densities of all 12 residues of peptide CR20 was clear and well defined. Notably, the interaction of peptide CR20 with LpxA is secured by a majority of polar interactions some of which are mediated by water molecules. There are approximately 13 water molecules involved in water-mediated interactions or supporting the peptide in the LpxA active site by hydrogen bonding (Fig. 4), suggesting that in the absence of substrate the active site is highly solvated. Delivery of the acyl chain by ACP would displace the solvent in the active site of LpxA.

Acyl carrier protein is an important component in fatty acid and polyketide biosynthesis. Recently, crystal structures of LpxD, the second acyltransferase in the Lipid A pathway, in complex with intact acyl-ACP gave an unprecedented view of how ACP delivers acyl chain linked to prosthetic 4′-phosphopantethiene group among acyl transferases in the lipid A pathway^15^. LpxA and LpxD share significant structural homology (Fig. 6d,e). A superposition of LpxA-peptide CR20 with LpxD-acyl-ACP reveal conservation of the acyl-ACP binding site (Fig. 6d–f). In support of previous enzymatic evidence that revealed Peptide 920 is largely competitive with respect to ACP^11^, it appears that Peptide 920 would block access of ACP to its binding site on LpxA. Perhaps, these basic residues (H160, R258, Q161 R204, R205, N198) (Fig. 3) play a role in ACP binding. ACP is a highly acidic protein with an isoelectric point of 4.1 and a pH solubility of roughly 3.9^21^. It is likely that the charge complementarity between the LpxA and ACP interacting regions would be disrupted by inhibitory peptides. The binding site of CR20 and the other peptides presented herein could be targeted for rational design of antibiotics that could block acyl chain delivery by ACP.

Because small peptides do not cross membranes and are subjected to protease degradation, the use of peptide CR20 or the other inhibitory peptides as drugs, targeting cytoplasmic LpxA is unrealistic. However, we have mapped the space and location of important residues of LpxA, that if engage by small molecules could contribute efficiently to the inhibition of LpxA. Therefore, all the structural and biochemical information garnered from this study are promising starting points for the development of antimicrobials using structure-based drug design. The results of this study should provide useful information for the further development of molecules that target the essential lipid A enzyme, LpxA.

## Materials and Methods

### Sample Preparation for crystallization

LpxA was overexpressed and purified as previously described^11,19^. Briefly, LpxA was purified from BL21(DE3)/pLysE/pTO1. The construct pTO1 is a pET23c (Novagen) vector that contains the wild type *lpxA* gene. Cells were lysed by one passage through a French pressure cell at 18,000 psi and centrifuged at 10,000 x*g* for 20 min to remove cell debris. The supernatants were further centrifuged at 100,000 x*g* for 90 min to obtain soluble proteins. The purification scheme consists of Green19-Agarose (Sigma) affinity chromatography (pH 7.4), followed by Source Q ion-exchange chromatography (pH 8) and Superdex 200 gel filtration chromatography in 10 mM potassium phosphate buffer, pH 7, containing 250 mM NaCl^8,19,22^. The purity of LpxA was evaluated by SDS-PAGE, LpxA activity assays, and electrospray ionization mass spectrometry. Mass spectra analyses were performed on a QSTAR XL quadrupole time-of-flight tandem mass spectrometer (ABI/MDS-Sciex, Toronto, Canada) equipped with an electrospray source^23^. Peptide CR20 (NH_2_-WMLDPIAGKWSR-COOH) and other truncated peptides described in this work were prepared at the University of North Carolina Peptide Synthesis Facility (Table 1). Each synthesized peptide was evaluated by MALDI mass spectrometry and was confirmed to have greater than 90% purity.

### Protein crystallization

Co-crystals of LpxA- peptide CR20 complex were obtained by combining a concentrated LpxA solution (20 mg/ml) with a 25-fold molar excess of peptide CR20 (12.5 mM). Crystals of LpxA- peptide CR20 were obtained by hanging drop vapor diffusion method from a 1:1 mixture of peptide and protein. Individual droplets contained 2 μl of the LpxA- peptide CR20 mixture and 2 μl of 0.8–1.8 M phosphate buffer, pH 6.3–6.9. Crystals appeared after 24 hrs and grew to approximately 1.0 mm after 2 weeks.

### Data Collection, Structure Determination and Refinement

Crystals of the LpxA-peptide CR20 complex were cryo-protected in 1.0 M Na/K phosphate, pH 6.9, and 35% DMSO, and then were flash cooled in liquid nitrogen. Diffraction data were collected on an R-Axis IV image plate detector. Diffraction images were processed and scaled using HKL2000. Crystals diffract to 1.60 Å and belong to the cubic space group P2_1_3 (a = b = c = 96.73 Å, Table 2). Phases were calculated using molecular replacement with the program MolRep in the CCP4i suite^24^. Previously published structure of LpxA monomer was used as the search model (PDB code 1LXA). Iterative rounds of model building were performed using O and COOT, with rounds of refinement in REFMAC^24–26^. The quality of the final model was evaluated using MolProbity and PROCHECK (PDB ID: 6HY2)^27,28^. The figures were drawn using PyMOL (DeLano Scientific, San Carlos, CA). Data collection and refinement statistics are presented in Table 2.

### Inhibition of LpxA activity by Peptide 920

The LpxA enzymatic reaction monitors the conversion of [α-^32^P]UDP-GlcNAc to [α-^32^P]UDP-3-O-(*R*-3-hydroxymyristoyl)- GlcNAc. The assay components consist of 40 mM HEPES buffer, pH 8, 1 mg/ml BSA, 1 μM *R*-3-hydroxymyristoyl-ACP, and 1 μM [α-^32^P]UDP-GlcNAc (2 × 10^6^ cpm/nmol). Truncated peptides (Table 1) was dissolved in DMSO and pre-incubated with the reaction mixture at 30 °C for 3 minutes in the absence of enzyme at concentrations ranging from 1 nM to 10 µM. The final concentration of DMSO was adjusted to 10% to match the peptide solvent in all enzymatic assays.

The reactions were started by the addition of 1 nM of LpxA and incubated at 30 °C for a maximum of 10 min. Three or four time points were collected during this time. Termination of the reaction was accomplished by the addition of 1 μl portions onto a silica thin layer chromatography (TLC) plate. The TLC plates were air dried for 10 min before developing in chloroform/methanol/water/acetic acid (25:15:4:2, v/v). Plates were exposed to PhosphorImager screens overnight, and the data were evaluated with Molecular Dynamics PhosphorImager equipped with ImageQuant software. Inhibition of LpxA by Peptide 920 and truncated peptides was analyzed by plotting the initial velocities as a function of the inhibitor concentration. To determine the IC_50_ at 30 °C, the data were fit to the following equations,

1 $${v}_{i}/{v}_{c}={{\rm{IC}}}_{50}/({\rm{I}}+{{\rm{IC}}}_{50})$$

or

1a $$ \% \,{\rm{activity}}=100/(1+{\rm{I}}/{{\rm{IC}}}_{50})$$

*v*_*i*_ represents the initial rate, at given concentrations of inhibitor, *v*_*c*_ represents the initial velocity of the control reaction without inhibitor, and I represents the inhibitor concentration. The IC_50_ determination represents the concentration of inhibitor needed to inhibit 50% of the enzyme activity.
